# Assessment of Thyroid Dysfunction Among Pregnant Women With Pre-Existing Diabetes Mellitus or Gestational Diabetes Mellitus

**DOI:** 10.7759/cureus.44390

**Published:** 2023-08-30

**Authors:** Eman A Alotaibi, Adhwa M AlHaidar, Shahad A Alotaibi, Norah A Alshehri, Raghad A Alotaibi, Yaser Y Bashumeel, Reema Nassar, Mohammed A Batais

**Affiliations:** 1 Department of Family and Community Medicine, Unaizah College of Medicine and Medical Sciences, Qassim University, Unaizah, SAU; 2 Primary Health Care, Saudi Ministry of Health, Riyadh, SAU; 3 College of Medicine, Sulaiman Al Rajhi University, Al Bukayriah, SAU; 4 Department of Family and Community Medicine, College of Medicine, King Saud University, Riyadh, SAU; 5 Research, Endocrine and Oncology Division, Department of Surgery, Tulane University School of Medicine, New Orleans, USA; 6 College of Medicine, Omdurman Islamic University, Khartoum, SDN; 7 Family Medicine, Diabetes & Chronic Disease Management, King Khalid University Hospital, Riyadh, SAU

**Keywords:** gestational diabetes mellitus (gdm), pregnant, thyroid dysfunction, gestational diabetes mellitus, diabetes mellitus

## Abstract

Objective

This study investigates the prevalence and risk of thyroid disturbances in pregnant women with pre-existing diabetes mellitus (DM) or gestational diabetes mellitus (GDM) in a tertiary hospital setting in Riyadh, SA. This research's findings may help identify potential risk factors associated with thyroid disturbances during pregnancy and facilitate early diagnosis for at-risk pregnant women.

Subjects and methods

A retrospective cross-sectional study was conducted at an endocrinology clinic between October 2018 and December 2021 to evaluate the electronic records of pregnant women with DM or GDM who had documented normal thyroid function before pregnancy.

Results

Three hundred ninety-six files that met the selection criteria were deeply investigated and analyzed. The analysis showed that 378 (95.5%) patients were of Saudi nationality, and the mean age in years ± SD for the selected patients was 34.23 ± 5.468. The prevalence of obesity was 63.7%, with a mean body mass index (BMI) of 32.78 ± 6.78 kg/m2.

The patients in this study were categorized into three groups based on their type of DM: 57 were diagnosed with type 1 DM (14.4%), 120 with type 2 DM (30.3%), and 219 with GDM (55.3%). The study identified 43 patients (10.85%) with subclinical hypothyroidism and 74 (18.69%) with hypothyroidism. Among the remaining patients, thyroid function was within the normal range for 264 (66.67%). The study also identified eight patients (2.02%) with subclinical hyperthyroidism and seven (1.77%) with hyperthyroidism. The prevalence of thyroid dysfunction was reported at 33.4%, with most of the dysfunction observed in the GDM group (20.7%). By comparison, the type 1 DM and type 2 DM groups presented a lower prevalence of thyroid dysfunction, accounting for only 4.1% and 8.6%, respectively.

Conclusions

Hypothyroidism, both clinical and subclinical, is more prevalent among patients with GDM than individuals with type 1 and type 2 DM. Research suggests a greater risk of developing hypothyroidism in patients with an increased BMI and among those older during pregnancy.

## Introduction

The thyroid is one of the organs of the endocrine systems in the human body and can be impacted by sustained high blood sugar levels and the body's constant attempts to eliminate this carbohydrate imbalance. Diabetes and thyroid dysfunction have been found to coexist in studies, with thyroid disease affecting glucose metabolism and untreated thyroid dysfunction impacting the management of diabetes. Many studies have highlighted an association between several types of diabetes and hypothyroidism; however, the evidence supporting this association is inconsistent [1,2]. However, certain risk factors for developing TD have been identified in patients with type 2 DM (T2DM), including body mass index (BMI), age, family history, smoking, parity, and pregnancy [1]. Pregnancy can significantly impact the body’s hormonal balance, particularly the thyroid gland. In pregnancy’s early stages, thyroid hormone levels may initially decrease before returning to normal levels as the pregnancy progresses. This reflects an increase in free thyroxine (FT4) under the influence of human chorionic gonadotropin (HCG) following a decrease in serum FT4 by approximately 10% to 15% [3]. Consequently, thyroid hormone and iodine requirements increase [4]. Moreover, TD and DM are common during pregnancy [5].

In Saudi Arabia, the prevalence of hypothyroidism among pregnant women is as high as 9.3% [6]-a serious concern. Hypothyroidism can adversely affect pregnancy outcomes, potentially leading to fetal death, pregnancy loss, low birth weight, and neurocognitive delays, which can lead to a lower intelligence quotient [4,7,8]. Moreover, among pregnant women worldwide, Saudis experience high rates of gestational diabetes mellitus (GDM) and pre-GDM [9]. GDM can lead to miscarriages, birth trauma, macrosomia, and other complications [7]. Consequently, pregnant women with GDM developing hypothyroidism could increase the risk of fetal and maternal complications.

A study in India concluded that women with GDM and hypothyroidism require intense surveillance and screening for other comorbidities, as they face an increased risk of perinatal complications [2]. In addition, several studies worldwide have shown that overt, subclinical hypothyroidism and anti-thyroid peroxidase (anti-TPO) are more prevalent among the pre-GDM group [10] and GDM group [11-14]. These findings indicate that routine screening is necessary for all patients with DM or an increased risk of GDM. However, some studies have not observed an increased risk of TD and GDM while still noting a pre-GDM risk [12, 15]. In light of these inconsistent results, this study aimed to further investigate the relationship between GDM/pre-GDM and the risk of developing TD in pregnancy.

## Materials and methods

A retrospective cross-sectional study was conducted in the endocrinology clinic at King Saud University Medical City (KSUMC), Riyadh, SA. Files of pregnant females who visited the clinic between October 2018 and December 2021 were accessed through the electronic records after approval from the Institutional Review Board of the research unit at King Saud University Medical City (project no. E-21-6025). A total of 1233 files were evaluated, of which 596 files (48.3%) correspond to patients with DM or GDM.

GDM was diagnosed per ADA recommendations using a one-step strategy of a 75-g OGTT. The diagnosis was made at 24-28 weeks of gestation when the plasma glucose measurement was equal to or exceeded 92 mg/dL (5.1 mmol/L) after fasting for at least eight hours; plasma glucose measurement was equal to or exceeded 180 mg/dL (10.0 mmol/L) at one hour; or equal to or exceeded 153 mg/dL (8.5 mmol) two hours postprandially. In between the 596 files of patients with DM or GDM, 396 files (32.1%) were related to patients with known normal thyroid functions. The other 200 files (16.2%) belong to patients with hypothyroidism (14.9%), hyperthyroidism (0.32%), subclinical hypothyroidism (0.81%), and patients who have undergone thyroidectomy (0.16%). Thyroid profiles were evaluated according to the guidelines of the American Thyroid Association (ATA) with trimester-dependent reference ranges: first trimester, 0.1-2.5 IU/mL; second trimester, 0.2-3.0 IU/mL; and third trimester, 0.3-3.0 IU/ mL". Subjects were excluded if they had any history of endocrine disorders before pregnancy, including thyroid dysfunction and Cushing disease, as it may influence fasting plasma glucose (FPG) levels. The main goal of the current study was to evaluate the risk of thyroid disturbances in pregnant patients with DM or GDM and known normal thyroid functions before pregnancy. All the collected data was kept confidential and used only for research purposes according to the principles of the Declaration of Helsinki for medical research.

Statistical analysis

The collected data were computerized, summarized, and statistically analyzed using IBM Corp. Released 2015. IBM SPSS Statistics for Windows, Version 23.0. Armonk, NY: IBM Corp. Qualitative parameters such as the family history of thyroid disorders, time of testing thyroid functions, type of thyroid disorder, Tg Ab, TPO Ab, body mass index (BMI), family history of DM, type of DM, history of comorbidities, neonatal complications, maternal complications, and history of other problems were displayed as frequencies out of the total. Moreover, quantitative parameters such as age, weight, height, number of children, gestational age, TSH, and T4 levels were presented as mean ± SD in different categories of thyroid disorders. ANOVA and Tukey’s post hoc tests were used to compare different types of thyroid disorders regarding all other parameters. Nonparametric Pearson’s correlation and linear regression between different items were calculated. The significance of the p-value was considered significant if it was less than 0.05.

## Results

**Figure 1:**
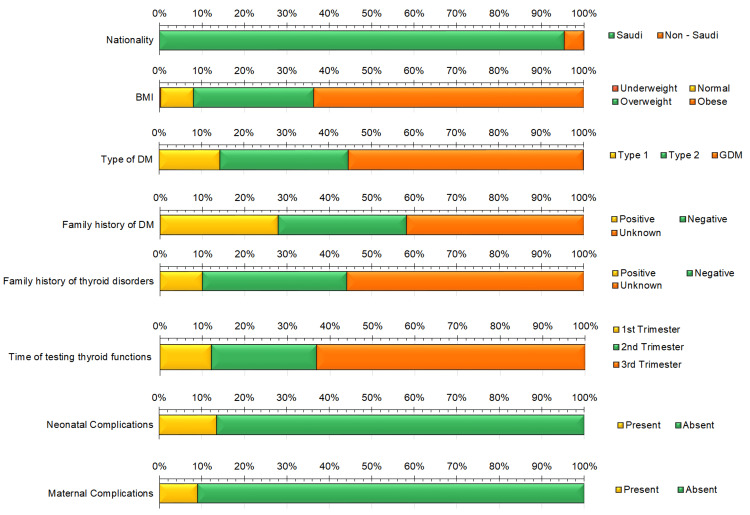
Frequencies of different calculated demographic characteristics. BMI: Body mass index. DM: Diabetes mellitus. GDM: Gestational diabetes mellitus.

The files of 396 women who met the selection criteria were analyzed. Among these women, 378 (95.5%) were of Saudi nationality, and 18 (4.5%) were non-Saudi. They had a mean age of 34.23 years (SD 5.468), a mean weight of 81.52 kg (SD 16.308), a mean height of 157.75 cm (SD 5.966), and a mean of 2.88 children (SD 1.993). Based on BMI, 252 of the women (63.6%) were obese, and 112 (28.3%) were overweight. Furthermore, 31 patients (7.8%) had normal BMIs, and one patient (0.3%) was underweight. Patients were classified into three groups: 57 patients with type 1 DM (14.4%), 120 with type 2 DM (30.3%), and 219 with GDM (55.3%). Three hundred and sixty-one patients (91.2%) reported no other associated health morbidities. However, 35 patients (8.8%) suffered from different health problems such as ulcerative colitis, colon cancer, Crohn’s disease, thrombophilia, bronchial asthma, and hypertension. Additionally, 268 out of 396 patients (67.7%) reported no history of maternal or fetal complications; 92 (23.2%) reported a history of maternal or fetal problems such as pre-eclampsia, placental abruption, and macrosomia that necessitated emergent delivery through cesarean section; and the remaining 36 patients (9.1%) reported a history of some fetal problems such as microsomia and intrauterine growth retardation (IUGR) and were managed by normal delivery. The records also revealed that the timing of thyroid function testing varied among pregnant patients. Thyroid functions were estimated in 48 patients (12.2%) during the first trimester, in 98 patients (24.7%) during the second trimester, and in 250 patients (63.1%) during the third trimester. All the measured frequencies, family history of DM or thyroid disorders, and simultaneous maternal or fetal complications are presented in Figure 1.

**Figure 2:**
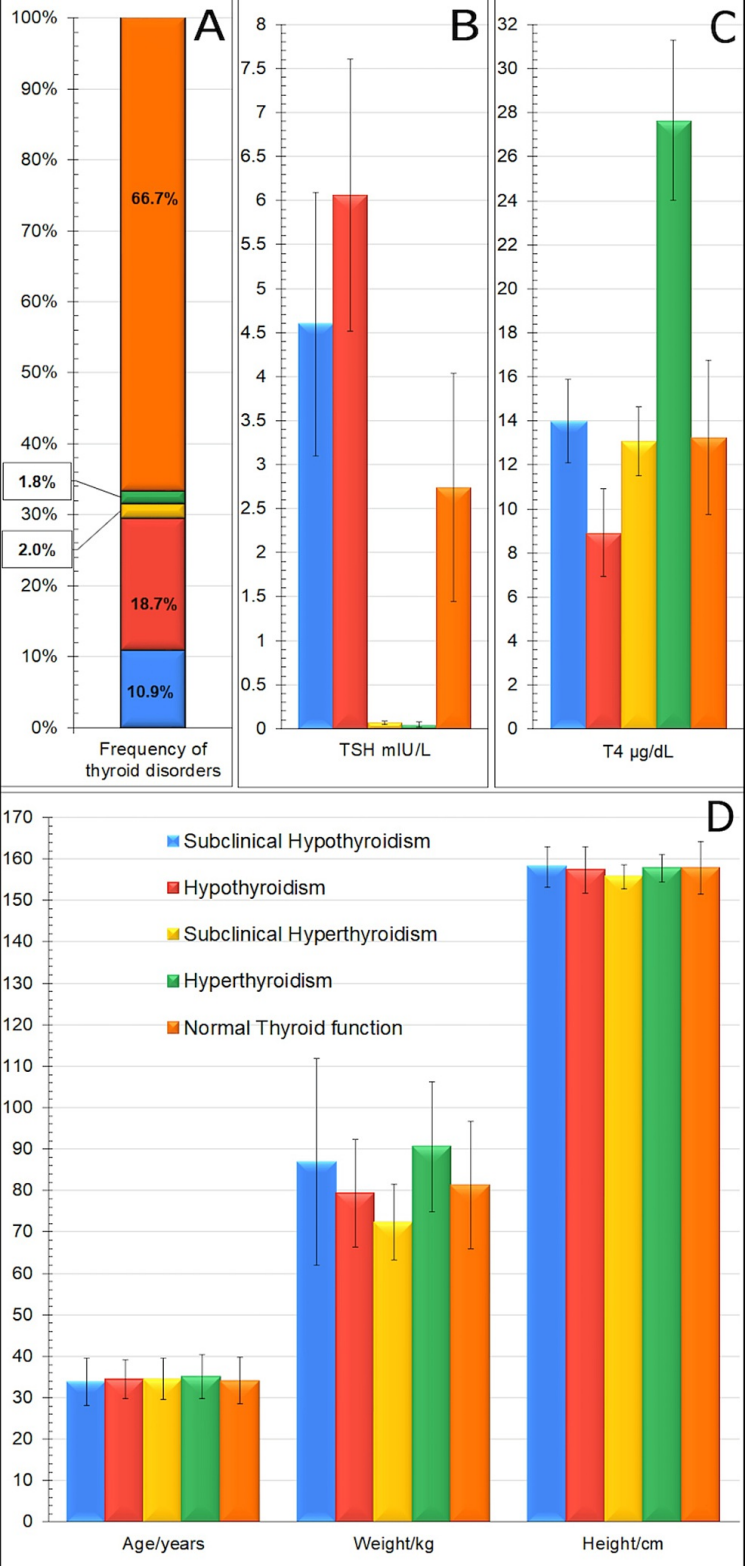
Frequencies of different thyroid disorders and the means ± SD of different measured parameters in each disorder.

In Figure 2, the frequency of thyroid disorders appears in Part A, in Part B the TSH level, in Part C the T4 level, and in Part D the rest of the demographic data such as weight, height, and age. The diagnosis of thyroid dysfunction was elaborated following ATA guidelines through estimation of TSH and T4 levels, with 43 patients (10.85%) diagnosed with subclinical hypothyroidism and 74 patients (18.69%) with hypothyroidism. Notably, eight (2.02%) and seven (1.77%) patients were diagnosed with subclinical hyperthyroidism and hyperthyroidism, respectively, while the thyroid functions in the remaining 264 patients (66.67%) were found to be normal. The means ± SD for the TSH levels, T4 levels, age, weight, and height in each group of patients classified according to thyroid functions are presented in Figure 2.

The distribution of frequencies regarding different parameters among groups, classified according to thyroid functions, is presented in Table 1.

**Table 1:** Frequencies of different parameters distributed among different thyroid disorders. Data are presented in each cell as numbers and (%). BMI: Body mass index, GDM: Gestational diabetes mellitus, HTN: Hypertension, IUGR: Intrauterine growth restriction.

Parameter		Subclinical Hypothyroidism Number (%) 43 (10.85)	Hypothyroidism Number (%) 74 (18.69)	Subclinical Hyperthyroidism Number (%) 8 (2.02)	Hyperthyroidism Number (%) 7 (1.77)	Normal Thyroid function Number (%) 264 (66.67)
BMI	Underweight	1 (0.3)	-	-	-	-
	Normal	2 (0.5)	6 (1.5)	1 (0.3)	-	22 (5.6)
	Overweight	12 (3.0)	19 (4.8)	3 (0.8)	1 (0.3)	77 (19.4)
	Obese	28 (7.1)	49 (12.4)	4 (1.0)	6 (1.5)	165 (41.7)
Nationality	Saudi	40 (10.1)	72 (18.2)	6 (1.5)	7 (1.8)	253 (63.9)
	Non - Saudi	3 (0.8)	2 (0.5)	2 (0.5)	-	11 (2.8)
Type of DM	Type-I	7 (1.8)	7 (1.8)	2 (0.5)	-	41 (10.4)
	Type-II	11 (2.8)	19 (4.8)	2 (0.5)	2 (0.5)	86 (21.7)
	GDM	25 (6.3)	48 (12.1)	4 (1.0)	5 (1.3)	137 (34.6)
Family history of DM	Positive	6 (1.5)	26 (6.6)	1 (0.3)	3 (0.8)	74 (18.7)
	Negative	13 (3.3)	21 (5.3)	4 (1.0)	2 (0.5)	80 (20.2)
	Unknown	24 (6.1)	27 (6.8)	3 (0.8)	2 (0.5)	110 (27.8)
History of comorbidities	HTN	2 (0.5)	3 (0.8)	1 (0.3)	-	10 (2.5)
	Others	2 (0.5)	7 (1.8)	1 (0.3)	1 (0.3)	8 (2.1)
	None	39 (9.8)	64 (16.2)	6 (1.5)	6 (1.5)	246 (62.1)
History of pregnancy and fetal problems	Macrosomia	1 (0.3)	-	-	-	3 (0.7)
	Microsomia	-	-	-	-	1 (0.3)
	IUGR	-	-	1 (0.3)	-	1 (0.3)
	CS	8 (2.0)	13 (3.3)	-	4 (1.0)	56 (14.0)
	Pre-eclampsia	-	-	1 (0.3)	-	2 (0.5)
	Placental abruption	-	-	-	-	1 (0.3)
	Others	6 (1.5)	4 (1.0)	2 (0.5)	-	24 (6.0)
	None	28 (7.1)	57 (14.4)	4 (1.0)	3 (0.8)	176 (44.4)
Family history of thyroid disorders	Positive	4 (1.0)	12 (3.0)	2 (0.5)	2 (0.5)	20 (5.1)
	Negative	14 (3.5)	23 (5.8)	3 (0.8)	2 (0.5)	93 (23.5)
	Unknown	25 (6.3)	39 (9.8)	3 (0.8)	3 (0.8)	151 (38.1)
Time of testing thyroid functions	1^st^ Trimester	3 (0.8)	7 (1.8)	2 (0.5)	-	36 (9.1)
	2^nd^ Trimester	11 (2.8)	23 (5.8)	2 (0.5)	-	62 (15.6)
	3^rd^ Trimester	29 (7.3)	44 (11.1)	4 (1.0)	7 (1.8)	166 (41.9)
Neonatal complications	Present	8 (2.0)	9 (2.3)	-	1 (0.3)	36 (9.1)
	Absent	35 (10.2)	65 (19.0)	8 (2.3)	6 (1.8)	228 (66.7)
Neonatal complications	Present	5 (1.3)	7 (1.8)	-	-	24 (6.1)
	Absent	38 (9.6)	67 (16.9)	8 (2.0)	7 (1.8)	240 (60.0%)

A non-parametric Spearman’s correlation was conducted to determine the relationship between the variables. In doing this, it was found that a family history of DM has a significant, moderately positive correlation with a family history of thyroid disorders (p <0.001 and r = 0.568). The significant correlations between different variables are highlighted in the correlation matrix in Table 2.

**Table 2:** Significant correlation matrix (non-parametric Spearman’s correlation). Data are presented in each cell as the r-value above and the p-value below. Week correlation if r < 0.3, moderate correlation 0.3 ≤ r < 0.7, and strong correlation if r ≥ 0.7. A p-value was considered significant if it was less than 0.05. BMI: Body mass index, TSH: thyroid stimulating hormone.

Parameters		Age	Height	Weight	BMI	Number of Children	TSH	T4	Maternal complications	Type of DM	Family history of DM
Age	Correlation coefficient	-	0.118	-	0.183	0.466	-	-0.097	-	-	-
	P-value	-	0.019	-	<0.001	<0.001	-	0.045	-	-	-
Weight	Correlation coefficient	0.219	0.362	-	-	0.183	-	-	-	-	-
	P-value	<0.001	<0.001	-	-	<0.001	-	-	-	-	-
BMI	Correlation coefficient	-	-	0.696	-	0.201	-	-	-	-	-
	P-value	-	-	<0.001	-	<0.001	-	-	-	-	-
Type of DM	Correlation coefficient	-	-	-	-	0.112	-	-	-	-	-0.126
	P-value	-	-	-	-	0.026	-	-	-	-	0.012
TSH	Correlation coefficient	-	-	-	-	-	-	-0.451	-	-	-
	P-value	-	-	-	-	-	-	<0.001	-	-	-
Time of testing thyroid functions	Correlation coefficient	-	-	-	-	-	-	-	-	0.138	-
	P-value	-	-	-	-	-	-	-	-	0.006	-
Type of thyroid disorder	Correlation coefficient	-	-	-	-	-	-0.676 <0.001	0.362 <0.001	-	-	-
	P-value	-	-	-	-	-		-	-	-	-
Family history of thyroid disorders	Correlation coefficient	-	-	-	-	-	-	-	-	-0.130	0.568
	P-value	-	-	-	-	-	-	-	-	0.010	<0.001
Neonatal complications	Correlation coefficient	0.174	-	-	-	-	-	0.095	0.156	-	-
	P-value	0.001	-	-	-	-	-	0.048	0.002	-	-
Maternal complications	Correlation coefficient	0.128	-	-	-	0.131	-	-	-	-	-
	P-value	0.011	-	-	-	0.009	-	-	-	-	-
History of comorbidities	Correlation coefficient	-	-	-	-	-	-	-	-	0.119	-
	P-value	-	-	-	-	-	-	-	-	0.018	-
History of other problems	Correlation coefficient	-	0.116	-	-	-	-	-	-	-0.106	0.112
	P-value	-	0.021	-	-	-	-	-	-	0.035	0.025

Linear regression was performed to confirm the association between different parameters. The identified relations showed considerable diversity; multiple relations detected by Spearman’s correlation were emphasized by linear regression. A significant negative relationship between the type of DM and the level of T4 (p = 0.043 and β = −0.023) was observed. Moreover, a highly significant positive relationship between a family history of DM and a family history of thyroid disorders was affirmed (p < 0.001 and β = 0.461). In addition, a strong association between Tg Ab and TPO Ab was confirmed by regression (p < 0.001 and β = 0.81 3). By contrast, more than 20 relations were identified by correlation and then denied by regression as a significant, weak correlation between the type of DM and the time of testing thyroid functions (p = 0.006 and r = 0.1 38). Also, many significant relations between different parameters were elaborated after linear regression, such as the relevance between the time of testing thyroid functions and a family history of DM (p = 0.049 and β = 0.042). Significant regressions were extracted and shown in Table 3.

**Table 3:** Significant regression matrix (linear regression). Data are presented in each cell as β values above and p-values below. A p-value was considered significant if it was less than 0.05. The highlighted cells show a combined significant correlation and regression. BMI: Body mass index, TSH: thyroid stimulating hormone.

Dependent variables	Age	Height	Weight	BMI	Number of Children	TSH	T4	Maternal complications	Type of DM	Family history of DM
Age					1.221 <0.001					0.842 0.020
Weight		0.766 <0.001								
Height				-2.901 <0.001						
BMI			0.025 <0.001		0.030 0.039					0.077 0.038
Number of Children										-0.039 0.049
Family history of DM						-0.245 0.031				
TSH							-0.740 <0.001			
Time of testing thyroid functions										0.042 0.049
Type of thyroid disorder						-0.894 <0.001	-0.544 0.047			
Family history of thyroid disorders										0.461 <0.001
Neonatal complications	0.011 0.002							0.188 0.002		
Maternal complications					0.021 0.014					
History of other problems		0.037 0.027				-0.089 0.035				0.300 0.031

## Discussion

In the present study, the mean age of participants was 34.23 years, similar to a local study [1] and higher than in other studies, where the mean ages were 32.1 and 29.9 years old, respectively [16]. The mean ± SD age of thyroid dysfunctions among different groups was 34.54 ± 5.175, which coincided with the data reported by Ghamri et al. [1]. This finding also dovetailed with another study that reported a higher incidence of hypothyroidism and hyperthyroidism among older pregnant women [7]. Late-age marriages and late-age pregnancies may thus play a role in this finding. 

Due to the study’s retrospective design, BMI was entered at the first obstetric check-up, as pre-pregnancy BMI information was unavailable. Following the trend of increasing BMI reported by the World Health Organization (an obesity prevalence of 33.7% [17] and a projected increase to 78% in 2022) [18], the prevalence of obesity in our study was 63.7%. Moreover, the mean BMI (32.78 ± 6.78 kg/m2) was virtually the same as that reported in another local study [1]. This is a major concern for obesity-related complications in pregnancy and delivery. These complications include GDM, pregnancy-induced hypertension, pre-eclampsia, induction of labor, preterm labor, preterm birth, increased rates of cesarean section, postpartum hemorrhage, anemia, urinary tract infection, wound infection, prolonged pregnancy, shoulder dystocia, fetal macrosomia, perinatal death, congenital fetal disabilities, and admission to the neonatal intensive care unit [19-25]. In alignment with other studies, we found no correlation between TD and BMI [1]. 

We found the prevalence of GDM to be higher than that of pre-GDM, with 55.3% compared to 30.3% in T2DM and 14.4% in T1DM. A similar dominance (81.25%) of the GDM group was found in a study carried out by Konar et al. [26]. In contrast, a separate study reported that only 36% of patients had GDM, but this may be attributable to the small sample size [27]. In our study, the prevalence of TD was 33.4%, mainly comprising clinical and subclinical hypothyroidism (29.6%). A similar pattern of results was obtained from another study that showed TD to be present in 40.62% with hypothyroidism predominance (37.5%) [26]. The present study demonstrated that 20.7% of GDM cases had thyroid dysfunctions, compared with 4.1% and 8.6% in T1DM and T2DM, respectively. Contrasted with another study [26], we found a higher rate of TD in GDM patients, which also differs from another study [27] that reported a significantly higher rate of TD in the pre-GDM group than in the GDM group. The cause of this difference may be attributed to the high prevalence of GDM and the exclusion of pregnant women with pre-GDM and known TD. 

A retrospective study conducted by Izzo et al. [28] examined patients with GDM to evaluate the association between GDM and thyroid dysfunction, finding that GDM patients are at greater risk of both clinical and subclinical hypothyroidism-a result that accords with that of the current study. This finding is crucial to understanding the relationship between TD and GDM. Hormones like estrogen, thyroid-binding globulin, HCG, placental lactogen, cortisol, and placental insulin enzyme affect blood glucose levels and maternal thyroid function during pregnancy [29,30]. Some studies have shown that there is a correlation between thyroid disease and GDM [31-35], whereas others have not reported this association [36,37]. Additionally, a meta-analysis supports this relation, showing that the incidence of GDM in patients with subclinical hypothyroidism was 1.35-fold higher than in the control group [38]. Although the prevalence of TD in GDM patients was high, no significant difference in thyroid function between the two groups (GDM and pre-GDM) was found, coinciding with the results of Konar et al. [26]. This lack of difference contrasts with the other study’s finding of a significantly higher rate of TD in patients with pre-GDM than in those with GDM [27]. 

Limitations and strengths: Given this study’s design, it is essential to note that a detailed history of participants and some lab results were not obtained. Unfortunately, due to the lack of TgAb and TPOAb, we could not investigate the autoimmunity factor associated with the relationship between DM and TD. Additionally, we did not have a control group with which to compare the prevalence of TD in healthy pregnant women to our population, limiting the broader applicability of our findings.

Despite these limitations, our study’s total participation was higher than in other studies on the same topic. This comparatively high participation rate reflects the reliability and accuracy of our results. Finally, it is relevant to note that we did not only investigate GDM but also included pre-GDM and further classified them into type 1 and type 2 DM, allowing for diversity in our results and enabling us to compare the effect of TD on each group. 

## Conclusions

Clinical and subclinical hypothyroidism are each more frequently observed in GDM patients than in those with type 1 and 2 DM. Further studies have indicated that pregnant patients with a higher BMI and advanced age are at greater risk of developing hypothyroidism during pregnancy. While there was no substantial discrepancy in TD between the GDM and pre-GDM groups, evaluating thyroid function in the high-risk group is highly advised because early prevention and management of TD can help to avoid fetal and maternal complications and enhance perinatal outcomes.
